# Haemoparasites of free-roaming dogs associated with several remote Aboriginal communities in Australia

**DOI:** 10.1186/1746-6148-8-55

**Published:** 2012-05-14

**Authors:** Emily N Barker, Debra A Langton, Chris R Helps, Graeme Brown, Richard Malik, Susan E Shaw, Séverine Tasker

**Affiliations:** 1 School of Veterinary Sciences, University of Bristol, Langford, Bristol BS40 5DU, UK; 2 Langford Veterinary Services, University of Bristol, Langford, Bristol BS40 5DU, UK; 3 Faculty of Veterinary Science, University of Sydney,, B14, NSW, Sydney 2006, Australia; 4 Centre for Veterinary Education, University of Sydney, Sydney, B22, NSW 2006, Australia

## Abstract

**Background:**

Tick-borne haemoparasites *Babesia vogeli* and *Anaplasma platys* are common among the free-roaming canine populations associated with Aboriginal communities in Australia, whilst the prevalence of haemoplasmas, which are also suspected to be tick-borne, remained unexplored. The aim of this study was to determine the prevalence of haemoplasma infection in these populations, and to identify any correlation with other haemoparasites. Blood was collected from 39 dogs associated with four Aboriginal communities and screened for infection using PCR and serology. DNA was purified and PCR analyses for piroplasms, *Anaplasmataceae* family bacteria and haemoplasmas performed. Serum was analysed using a commercial haemoparasite ELISA. Prevalence of infection was compared between communities.

**Results:**

Seventeen dogs (44%) were infected (PCR positive) with *Mycoplasma haemocanis*, eight (21%) with ‘*Candidatus* Mycoplasma haematoparvum’, 20 (51%) with *A. platys*, and 17 (44%) with *B. vogeli*. Two dogs were infected with a novel haemoplasma as determined by DNA amplification and sequencing. Two dogs (5%) were serologically positive for *Dirofilaria immitis* antigens, one (3%) was positive for *Ehrlichia canis* antibodies and nine (24nbsp;%) were positive for *A. platys* antibodies. Co-infections were frequent. Haemoplasma prevalence was highest (73%, 16/22) in Central Australia and lowest (22%, 2/9) in Western Australia (p = 0.017). In contrast, *B. vogeli* prevalence was low in Central Australia (18%, 4/22) but higher (78%, 7/9) in Western Australia (p = 0.003).

**Conclusions:**

This is the first time haemoplasma infections, including a novel species, have been molecularly documented in Australian dogs. The wide regional variation in prevalence of some of the haemoparasite infections detected in this study warrants further investigation.

## Background

Dogs associated with the Aboriginal communities in the remote regions of Australia are considered to be free-roaming as they are owned but not confined in any manner. Amongst this canine population, infestation with the brown dog tick (*Rhipicephalus sanguineus*) is endemic and infections with *Anaplasma platys* and *Babesia vogeli,* tick-borne haemoparasites associated with *R. sanguineus,* are widespread [1]. The canine haemoplasmas *Mycoplasma haemocanis* and ‘*Candidatus* Mycoplasma haematoparvum’ are also believed to have *R. sanguineus*-mediated transmission [2], with free-roaming behaviour suspected of being a significant risk factor for the relatively high haemoplasma prevalence (20%) in Tanzanian dogs [3]. A recent PCR-based study of a canine hospital population in Sydney, New South Wales did not identify any haemoplasma infections (N. Hetzle, personal communication), although the blood was collected from non free-roaming owned dogs in a large city where the brown dog tick is very rarely found.

**Figure 1:**
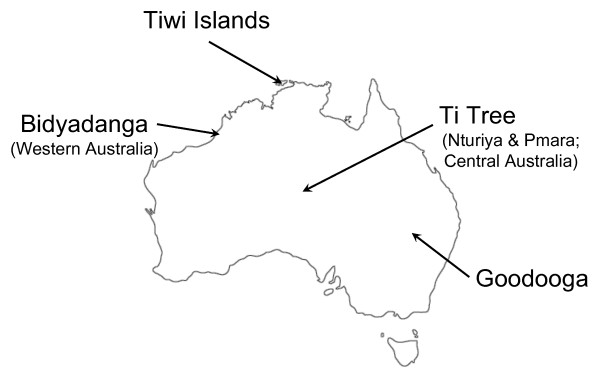
Map of Australia with sampling sites indicated.

The aim of this study was to determine the prevalence of canine haemoplasma infections in free-roaming dogs from several remote Aboriginal communities (see Figure 1), Ti Tree (Nturiya and Pmara) in the Northern Territory (Central Australia), Tiwi Islands (off the coast of the Northern Territory), Goodooga (north-western New South Wales) and Bidyadanga (Western Australia), using real-time quantitative PCR analysis. A further aim was to determine if such infections were associated with other haemoparasites.

## Results

### Population

Of the 39 dogs sampled, 20 were male and 19 were female (Table 1). Most of the dogs were entire. The majority of dogs were adults (n = 25), with equal numbers (n = 7) of puppies and juvenile dogs.

**Table 1:** **Population data, PCR and *A. platys *serological results for each Aboriginal community**

	**Population**			**PCR**				**Serology**
**Aboriginal community**	**Dogs sampled**	**Age (puppy / juvenile / adult)**	**Gender (M/F)**	***Mycoplasma haemocanis***	**‘*Ca*. Mycoplasma haematoparvum’**	***Anaplasma platys***	***Babesia vogeli***	***Anaplasma platys***
**Ti Tree**^**§**^**:**	22	1/5/16	13/9	14 (63.6%)*	7 (31.8 %)*	8 (36.4%)	4 (18.2 %)	5 (23.8%)
**Nturiya**^**†**^	11	0/3/8	8/3	5 (45.5%)	3 (27.3%)	4 (36.4%)	1 (9.1%)	1 (9.1%)
**Pmara**	11	1/2/8	5/6	9 (81.8%)	4 (36.4%)	4 (36.4%)	3 (27.3%)	4 (40.0%)^#^
**Tiwi Islands**	3	0/1/2	1/2	1 (33.3%)	0 (0%)	3 (100%)	3 (100%)	1 (33.3%)
**Goodooga**	5	1/0/4	2/3	1 (20.0%)	1 (20.0%)	2 (40.0%)	3 (60.0%)	0 (0%)
**Bidyadanga**^**†**^	9	5/1/3	4/5	1 (11.1%)	0 (0%)	7 (77.8%)	7 (77.8%)	3 (33.3%)
**Total**	**39**	**7/7/25**	**20/19**	**17 (43.6%)**	**8 (20.5%)**	**20 (51.3%)**	**17 (43.6%)**	**9 (23.7%)**

The number (and percentages, in parentheses) of dogs infected with each haemoparasite is recorded.

^**§**^ The Ti Tree results are the sum of those from Nturiya and Pmara.

*Six dogs were co-infected with both *M. haemocanis* and ‘*Ca*. M. haematoparvum’.

^†^The novel haemoplasma was detected in two dogs: one from Nturiya (Ti Tree) and one from Bidyadanga.

^#^Data available for 10 of the 11 dogs.

### PCR Results

PCR results were available for all samples for all assays. DNA was successfully purified and amplified from all canine blood samples as determined by the presence of adequate levels of canine internal control gene glyceraldehyde 3-phosphate dehydrogenase (GAPDH; threshold cycle: 18.2 to 23.3).

See Table 1 for a complete breakdown of results for each region (See Additional file 1: Table S1 for results of individual dog). Of the 39 dogs, 17 (44%) were infected, as determined by a positive quantitative PCR (qPCR) result, with *M. haemocanis* (threshold cycle: 22.3 to 38.2) and eight (21%) with ‘*Ca*. M. haematoparvum’ (threshold cycle: 27.2 to 35.2). Twenty dogs (51%) were infected with *A. platys* (threshold cycle: 24.4 to 41.3) as determined by qPCR, 16 of these dogs were also positive using the *Anaplasmataceae* family conventional PCR (cPCR). No samples were positive by the *Anaplasmataceae* family cPCR and negative by the *A. platys* qPCR. Amplicons from two positive samples using the *Anaplasmataceae* family cPCR assay were confirmed by sequencing to contain *A. platys* DNA. Seventeen (44%) of the 39 dogs were positive, as determined by cPCR, for a *Babesia*/*Theileria* spp. piroplasm. Amplicon sequencing of 15 of the 17 positive results indicated the presence of *B. vogeli* (insufficient amplification of target DNA from the remaining two positive samples resulted in a failure to sequence).

**Figure 2:**
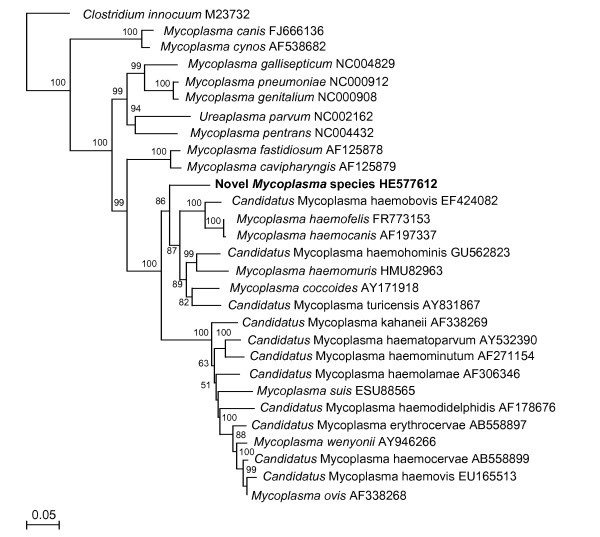
**Phylogenetic analysis of partial 16 S ribosomal gene sequences for the newly described haemoplasma species (shown in bold), other available haemoplasma species and selected non-haemoplasma *Mycoplasma *species.** The phylogenetic tree was rooted to *Clostridium innocuum * [GenBank: M23732]. The tree was constructed by the neighbour-joining method. Evolutionary distances are to the scales shown. GenBank accession numbers are shown for all sequences.

All dogs positive for *M. haemocanis* and/or ‘*Ca*. M. haematoparvum’ were also positive by the pan-haemoplasma assay, but two additional dogs were positive only on the pan-haemoplasma assay (threshold cycle: 36.7 & 37.9), corresponding to approximately one to ten haemoplasmas per reaction (equivalent to 5 μl DNA template); one from Nturiya (Ti Tree) and one from Bidyadanga. Of these dogs, one was negative for all other haemoparasites, and the other was positive for *A. platys* and *B. vogeli*. Sequencing of a 600 bp 16 S rRNA gene fragment amplified from these discordant samples showed that they were both infected with the same novel haemoplasma species [EMBL:HE577612] (Figure 2: phylogenetic tree). The novel haemoplasma had highest identity (82.8 to 84.9%) to the haemofelis group of haemoplasmas [4], closely followed by the haemominutum group of haemoplasmas (79.1 to 83.7%) and the non-haemotropic mycoplasmas *Mycoplasma fastidiosum* and *Mycoplasma cavipharyngis* (82.3 and 82.5% respectively). The 23 bp deletion common to haemofelis group haemoplasmas was not present in the novel haemoplasma gene sequence. Attempts to amplify a larger 16 S rRNA gene fragment and a ribonuclease P ribosomal gene fragment from these two samples were unsuccessful.

### Serology

Serological results were available for all dogs, except for an *A. platys* antibody result for the only dog (from Pmara, Ti Tree) that was positive for *E. canis* antibodies. Nine dogs (23.7%) were positive for *A. platys* antibodies (Table 1). Both dogs that were serologically positive for *D. immitis* antigen were from Nturiya. No dogs were positive for *B. burgdorferi* antibodies.

### Co-infections & concurrent seropositive results

Six of the dogs in Ti Tree were infected with both *M. haemocanis* and ‘*Ca*. M. haematoparvum’, whilst co-infection with *M. haemocanis* and ‘*Ca*. M. haematoparvum’ was not present in the other communities. Six of the haemoplasma positive dogs were infected with *A. platys* as determined by qPCR: one was infected with *M. haemocanis* alone; two with ‘*Ca*. M. haematoparvum’ alone; two co-infected with *M. haemocanis* and ‘*Ca.* M. haematoparvum’; and a further dog infected with the novel haemoplasma. Six dogs positive for *B. vogeli,* as determined by cPCR, were positive for one or more haemoplasma: three with *M. haemocanis* alone; one with ‘*Ca*. M. haematoparvum’ alone; one co-infected with *M. haemocanis* and ‘*Ca.* M. haematoparvum’; and a further dog with the novel haemoplasma. Three dogs infected with *M. haemocanis*, one of which was co-infected with ‘*Ca*. M. haematoparvum’, were seropositive for *A. platys*. One dog co-infected with *M. haemocanis* and ‘*Ca*. M. haematoparvum’ was also seropositive for *D. immitis* antigen.

Of the 20 dogs infected with *A. platys* as determined by qPCR, 13 were cPCR-positive for *B. vogeli*, six were seropositive for *A. platys* antibodies (one dog had an unknown result), one was seropositive for *E. canis* antibodies, and one was seropositive for *D. immitis* antigen.

Of the 17 dogs infected with *B. vogeli* as determined by cPCR, five were seropositive for *A. platys* antibodies (one unknown), one was serpositive for *E. canis* antibodies, and none were seropositive for *D. immitis* antigen.

The dog that was seropositive for *E. canis* antibodies was also PCR positive for *A. platys*, *B. vogeli*, *M. haemocanis* and ‘*Ca*. M. haematoparvum’.

### Comparisons between aboriginal communities

Due to the low numbers of dogs from each community, only haemoparasite prevalences from Central Australia (Ti Tree) and Western Australia (Bidyadanga) could be compared. Haemoplasma prevalence was highest (16/22; 73%) in Central Australia and lowest (2/9; 22%) in Western Australia. The difference in haemoplasma prevalence between these two sites was statistically significant (p = 0.017; Fisher's exact test). In contrast, *B. vogeli* prevalence was low in Central Australia (4/22; 18%) but higher (6/9; 66%) in Western Australia. The difference in *B. vogeli* prevalence between these two sites was statistically significant (p = 0.003; Fisher's exact test). There was a trend towards a statistically significant difference in *A. platys* prevalence between Central Australia (8/22; 36%) and Western Australia (7/9; 78%; p = 0.054; Fisher’s exact test).

## Discussion

Prevalence of the haemoparasites *A. platys* and *B. vogeli* in this report was higher (51.3% & 43.6% respectively; 33.3% dual *A. platys* &*B. vogeli* infection) than in earlier studies of a similar population of free-roaming dogs associated with Aboriginal communities, where 43% were positive for *A. platys* and 21% for *B. vogeli,* including 11% dual infections [1]. The study [1] also reported marked regional variation, with *A. platys* infection ranging from 39% in north-western New South Wales (including the Goodooga community) to 50% in coastal Arnhem Land in the Northern Territory (near the Tiwi Islands) and *B. vogeli* infection ranging from 9% in the Northern Territory to 29% in the Tanami desert (approximately half way between the Tiwi Islands and Ti Tree communities).

Canine haemoplasma prevalence outside of Australia has ranged from 0% in the UK [5] to 40% in Sudan [6], with higher prevalences tending to be seen in warmer climates. This compares to a haemoplasma prevalence rate of 54% in the free-roaming canine population described in this study. *Mycoplasma haemocanis* prevalence has been reported to be up to 40% [7] and ‘*Ca*. M. haematoparvum’ prevalence up to 33% [6] with co-infections identified in up to 17% of haemoplasma positive dogs [8], compared to figures of 44%, 21% and 32%, respectively from the present study. It was not possible to determine whether dogs in this study were co-infected with a *M. haemocanis* or ‘*Ca*. M. haematoparvum’ and the novel haemoplasma due to the methodology. In the authors’ opinion the less than 85% identity to known *Mycoplasma* species and the absence of the characteristic deletion common to the most closely related haemofelis group of haemoplasmas are sufficient to be able to describe the haemoplasma identified as being novel [9]. Unfortunately, copy numbers of the novel haemoplasma within the blood of both dogs identified as being infected were too low to enable molecular description sufficient to name the novel species. The significance and the primary host of the novel haemoplasma have yet to be determined.

Canine heartworm has been reported in domestic dogs in Southern Australia and dingoes in the Northern Territories, so the detection of *D. immitis* antigen was not unexpected [10,11]. It is uncertain and controversial as to whether Lyme borreliosis occurs in Australia [12]. Currently, there is no evidence that *B. burgdorferi* is responsible for a local syndrome, which has clinical features reminiscent of Lyme disease, so absence of seropositivity in dogs was to be expected. The positive serological result for *E. canis* could not be confirmed by *Anaplasmataceae* family cPCR, as the dog was also positive for *A. platys* by qPCR*.* In addition, false positive results for *E. canis* antibody tests have previously been reported in Australia, indicating the presence of cross-reacting antibodies [13], raising the suspicion of a false positive result in this case, however, exposure or co-infection with a low copy number of *E. canis* organisms could not be ruled out.

Inferences from the statistical analysis were limited by the small number of samples obtained in some of the Aboriginal communities, which in turn were limited by a short time-frame for sample collection, and the free-roaming nature of the dogs. It is hoped that future studies involving haematological analyses in conjunction with PCR-based detection of haemoparasites from a larger number of dogs from a single community will elucidate the potential role of canine haemoplasma infection and disease, as both *A. platys* and *B. vogeli* have been associated with a reduction in mean platelet numbers [1] while anaemia has been associated only with *B. vogeli*. The role of haemoplasmas and their mode of transmission in this setting need to be ascertained to determine whether interventions with antimicrobial and / or ectoparasiticidal agents are appropriate. Identification of additional animals infected with a greater copy number of the novel haemoplasma would be required to enable further characterisation and complete molecular description. A much greater number of samples from Tiwi Islands, Goodooga and Bidyadanga would be required to accurately assess the prevalence of *D. immitis* infection in these populations.

## Conclusions

Dogs living in aboriginal communities represent a naturally occurring model of polymicrobial haemoparasitic infection. Similar to *A. platys* and *B. vogeli* infection, haemoplasma infection, either with single species or co-infections was common in the free-roaming canine populations associated with Aboriginal communities. The wide variation in regional infection prevalence between some of the haemoparasite infections warrants further investigations to determine the role of vectors in disease transmission and the influence of concurrent haemoparasite infections on susceptibility and pathogenicity, especially in relation to the occurrence of anaemia and thrombocytopenia.

## Methods

### Samples

EDTA-blood and whole blood (clot tube) were collected from 39 free-roaming dogs associated with four Aboriginal communities, between February 2008 and October 2009. Blood was stored at 4°C, and transported to the laboratory within seven days of collection. Serum was harvested from whole blood after centrifugation. EDTA blood and serum were subsequently frozen at −70°C until required.

Most dogs were of mixed breed, and were considered variable hybrids of the dingo (*Canis lupus dingo*) and the domestic dog (*Canis lupus familiaris*). Based on dentition they were assigned to three age classes: puppy (< 3 months), juvenile (3 to 12 months) and adult (> 12 months).

### DNA extraction and PCR analysis

DNA was purified from the samples using the QIAmp® DNA Mini Kit (Qiagen Pty Ltd) according to the manufacturer’s recommendations, then shipped to the UK on dry ice.

Purified DNA was subjected to an *Anaplasmataceae* family conventional PCR cPCR and a *Babesia* / *Theileria* spp. cPCR. The *Anaplasmataceae* family cPCR comprised a primer pair (EHR16SD GGTACCYACAGAAGAAGTCC & EHR16SR TAGCACTCATCGTTTACAGC) [14], which amplifies a 345 base pair (bp) fragment from the 16 S rRNA gene using the following thermal protocol: 95°C for 10 min and 40 cycles of 95°C for 30 s, 55°C for 30 s, and 72°C for 30 s. The *Babesia*/*Theileria* spp. cPCR comprised a primer pair (BabgenF GAAACTGCGAATGGCTCATTA & BabgenR CGGTAGGCCAATACCCTACCGTC) [15], which amplifies a 250–270 bp fragment from the 18 S rRNA gene using the following thermal protocol: 94°C for 10 min and 40 cycles of 94°C for 30 s, 65°C for 30 s, and 72°C for 45 s. All reactions used 2x HotStar*Taq* Master Mix (Qiagen, Crawley, UK) with 200 nM each primer pair, 3.0 mM final MgCl_2_ and 2 μL DNA template in a total volume of 20 μL, and were performed in a Tetrad thermal cycler (MJ Research, Waltham, MA, USA). Positive and negative control reactions were used in each assay. Amplification products were visualised on an agarose gel following electrophoretic separation.

Purified DNA was also subjected to the following qPCR assays: species-specific qPCRs for each of *M. haemocanis* and ‘*Ca.* M. haematoparvum’, each duplexed with a GAPDH qPCR [3], pan-haemoplasma qPCRs [16], and an *A. platys*-specific qPCR (personnel communication: M Robinson, Acarus Laboratory, University of Bristol). Briefly, the canine species-specific qPCRs were performed using 2x HotStar*Taq* Master Mix with 200 nM species-specific haemoplasma 16 S rRNA gene primer pair (*M. haemocanis*: Mhf 1167 F GTGCTACAATGGCGAACACA & Mhf 1246R TCCTATCCGAACTGAGACGAA; ‘*Ca.* M. haematoparvum’: CMhp 124 F GGAGAATAGCAATCCGAAAGG & CMhp 252R GCATTTACCCCACCAACAAC), 100 nM haemoplasma *Taq*Man probe (*M. haemocanis*: Mhf 1188 T *FAM*-TGTGTTGCAAACCAGCGATGGT-*BHQ1*; ‘*Ca.* M. haematoparvum’: CMhp 192 T *FAM*-CTTCGGGAGCCCCGCGC-*BHQ1*), 25 nM canine GAPDH gene primer pair (17 F TCAACGGATTTGGCCGTATTGG & 106R TGAAGGGGTCATTGATGGCG), 50 nM canine GAPDH *Taq*Man probe (*TXR*-CAGGGCTGCTTTTAACTCTGGCAAAGTGGA-*BHQ2*), 4.5 mM final MgCl_2_ and 5 μl gDNA in a total volume of 25 μl. Briefly, the pan-haemoplasma qPCRs were performed using 2x HotStar*Taq* Master Mix with 200 nM haemoplasma 16 S rRNA gene primer pair (Haemofelis group: HF grp 567 F GGAGCGGTGGAATGTGTAG & HF grp 680R GGGGTATCTAATCCCATTTGC; Haemominutum group: HM grp 1061 F GGGGCCAAGTCAAGTCATC & HM grp 1199R GCGAATTGCAGCCTTTTATC), 100 nM haemoplasma *Taq*Man probe (Haemofelis group: HF grp 595P *FAM*-TYAAGAACACCAGAGGCGAAGGCG-*BHQ1*; Haemominutum group: HM grp 1096P *FAM*-TACCATTGTAGCACGTTYGCAGCCC*-BHQ1*), 4.5 mM final MgCl_2_ and 5 μl gDNA in a total volume of 25 μl. All haemoplasma qPCRs were performed in an Agilent MX3005P real-time PCR machine (Agilent Technologies UK Ltd., Wokingham, UK): 95°C for 15 min and 45 cycles of 95°C for 10 s and 60°C for 30 s, during which fluorescence data were collected. Briefly, the *A. platys*-specific qPCRs were performed using 2x HotStar*Taq* Master Mix (Qiagen, Crawley, UK) with 200 nM *A. platys*-specific citrate synthase gene (*gltA*) primer pair (APLgltA1.f AGGCGTGATTTCATCCTTCA & APLgltA1.r CACAGCAAGCTCTTCATTTCC), 100 nM *gltA Taq*Man probe (APLgltA1.p *FAM-*TGGCTGCGAAGTATCATGGGGA*-BHQ1*), 5.0 mM final MgCl_2_ and 5 μl gDNA in a total volume of 25 μl. All reactions were performed in an Opticon (Bio-Rad Labs. Ltd., Hemel Hempstead, UK) real-time PCR machine: 95°C for 15 min and 45 cycles of 95°C for 10 s and 64°C for 15 s, during which fluorescence data were collected. Positive and negative control reactions were used in each assay. Samples with discordant haemoplasma results (generic assay positive but *M. haemocanis* & ‘*Ca.* M. haematoparvum’ negative) were subjected to cPCR amplification using 2x HotStar*Taq* Master Mix, 200 nM of a universal *Mycoplasma* primer pair (HBT-F ATACGGCCCATATTCCTACG & HBT-R TGCTCCACCACTTGTTCA) [17], 3.75 mM final MgCl_2_ and 5 μL DNA template in a total volume of 25 μL, using the following thermal protocol: 95°C for 15 min and 50 cycles of 95°C for 10 s, 55°C for 15 s and 72°C for 30 s in a MJ Mini thermal cycler (Bio-Rad Labs. Ltd.).

Amplicons from positive cPCR results were purified using the NucleoSpin® Extract II Kit (Macherey-Nagel, ABgene, Epsom, UK), and subjected to DNA sequencing using Applied Biosystems Big-Dye Ver 3.1 chemistry on an Applied Biosystems model 3730 automated capillary DNA sequencer. BLASTn analysis [18] was performed to compare the 16 S rRNA gene (*Anaplasmataceae* family & haemoplasmas) and 18 S rRNA gene (*Babesia*/*Theileria* spp.) sequences obtained to those in GenBank. A phylogenetic tree including existing haemoplasma species, as well as selected non-haemoplasma *Mycoplasma* species, was constructed using MacVector version 12 (MacVector, Inc., Cambridge, United Kingdom) for the 16 S rRNA gene using the neighbour-joining program from a distance matrix [19], corrected for nucleotide substitutions by the Kimura two-parameter model [20]. The data set was re-sampled 1000 times to generate bootstrap percentages. The 16 S rRNA gene fragment of the novel haemoplasma species was deposited in the European Molecular Biology Laboratory Nucleotide Database (HE577612).

### Serology

Serum samples were tested by enzyme-linked immunosorbent assay (ELISA) for *Dirofilaria immitis* (heartworm) antigen and *Ehrlichia canis*, *Borrelia burgdorferi*, *Anaplasma phagocytophilum* antibodies using the SNAP 4Dx kit (Idexx Laboratories Pty. Ltd., Rydalmere, NSW 2116, Australia). Due to cross reactivity between *A. platys*- and *A. phagocytophilum*-directed antibodies, in conjunction with the absence of *A. phagocytophilum* from Australia, a positive *A. phagocytophilum* ELISA result was taken to indicate exposure to *A. platys* (Personal Communication: Idexx Laboratories Pty. Ltd.).

### Statistical analysis

Data were entered into Microsoft® Office Excel® 2007 (Microsoft Corporation) and statistical analyses performed using Statistical Package for the Social Sciences version 18.0 (SPSS Inc., Chicago, IL). Categorical data, i.e. absence or presence of specific infection, gender, age, Aboriginal community (Ti Tree & Bidyadanga only), were compared using the chi-square test, or the Fisher’s exact test where sample sizes were small. Too few dogs were available from Tiwi Islands and Goodooga to allow statistical evaluation of area influence on infection. Insufficient numbers were available in each category to enable multivariate analysis. Significance was taken as p ≤ 0.05.

### Animal ethics

All procedures were performed with the approval of the Animal Ethics committee of the University of Sydney (N00/11-2006/3/4492).

## Authors’ contributions

GB, RM, SS and ST conceived of the study. GB and RM collected the samples and performed the ELISAs. ENB, DL and CRH carried out the PCR screening assays, DNA sequencing and analysis. ENB, CRH and ST drafted the manuscript. All authors read and approved the final manuscript.

## Supplementary Material

Additional file 1 **Table S1.** For individual dogs: population data (Aboriginal community; age; sex); *Babesia vogeli* (Bv) conventional PCR (cPCR) results [positive or not-detected (n/d)], *Anaplasma platys* (Ap), *‘Candidatus* Mycoplasma haematoparvum’ (CMhp) and *Mycoplasma haemocanis* (Mhc) quantitative PCR (qPCR) results [threshold cycle for positive samples or not-detected (n/d)]; *Dirofilaria immitis* (Di), *Borrelia burgdorferi* (Bb), *Ehrlichia canis* (Ec), and *A. platys* (Ap) serological results. Click here for file

## References

[B1] Brown GK Canfield PJ Dunstan RH Roberts TK Martin AR Brown CS Irving R Detection of *Anaplasma platys* and *Babesia canis vogeli* and their impact on platelet numbers in free-roaming dogs associated with remote Aboriginal communities in Australia Aust Vet J 2006 84 9 321 325 10.1111/j.1751-0813.2006.00029.x 16958629

[B2] Seneviratna P Weerasinghe N Ariyadasa S Transmission of *Haemobartonella canis* by dog tick, *Rhipicephalus sanguineus* Res Vet Sci 1973 14 1 112 114 4736045

[B3] Barker EN Tasker S Day MJ Warman SM Woolley K Birtles RJ Georges K Ezeokoli CD Newaj-Fyzul A Campbell M Development and use of real-time PCR to detect and quantify *Mycoplasma haemocanis* and “*Candidatus* Mycoplasma haematoparvum” in dogs Vet Microbiol 2010 140 1–2 167 170 19646827 10.1016/j.vetmic.2009.07.006 PMC2805721

[B4] Peters IR Helps CR McAuliffe L Neimark H Lappin MR Gruffydd-Jones TJ Day MJ Hoelzle K Willi B Meli ML RNaseP RNA gene (*rnpB*) phylogeny of mycoplasmas and other *Mycoplasma* species J Clin Microbiol 2008 46 5 1873 1877 10.1128/JCM.01859-07 18337389 PMC2395117

[B5] Warman SM Helps CR Barker EN Day S Sturgess K Day MJ Tasker S Haemoplasma infection is not a common cause of canine immune-mediated haemolytic anaemia in the UK J Small Anim Pract 2010 51 10 534 539 10.1111/j.1748-5827.2010.00987.x 21029097

[B6] Inokuma H Oyamada M Davoust B Boni M Dereure J Bucheton B Hammad A Watanabe M Itamoto K Okuda M Epidemiological survey of *Ehrlichia canis* and related species infection in dogs in eastern Sudan Ann N Y Acad Sci 2006 1078 461 463 10.1196/annals.1374.085 17114754

[B7] Novacco M Meli ML Gentilini F Marsilio F Ceci C Pennisi MG Lombardo G Lloret A Santos L Carrapico T Prevalence and geographical distribution of canine hemotropic mycoplasma infections in Mediterranean countries and analysis of risk factors for infection Vet Microbiol 2010 142 3–4 276 284 19931320 10.1016/j.vetmic.2009.09.069

[B8] Kenny MJ Shaw SE Beugnet F Tasker S Demonstration of two distinct hemotropic mycoplasmas in French dogs J Clin Microbiol 2004 42 11 5397 5399 10.1128/JCM.42.11.5397-5399.2004 15528754 PMC525152

[B9] Drancourt M Raoult D Sequence-based identification of new bacteria: a proposition for creation of an orphan bacterium repository J Clin Microbiol 2005 43 9 4311 4315 10.1128/JCM.43.9.4311-4315.2005 16145070 PMC1234117

[B10] Starr TW Mulley RC *Dirofilaria immitis* in the dingo (*Canis familiaris dingo*) in a tropical region of the Northern Territory, Australia J Wildl Dis 1988 24 1 164 165 3352087 10.7589/0090-3558-24.1.164

[B11] Copland MD O'Callaghan MG Hajduk P O'Donoghue PJ The occurrence of *Dirofilaria immitis* in dogs in South Australia Aust Vet J 1992 69 2 31 32 10.1111/j.1751-0813.1992.tb07429.x 1632726

[B12] Russell RC Lyme disease in Australia - still to be proven! Emerg Infect Dis 1995 1 1) 29 31 8903152 10.3201/eid0101.950106 PMC2626827

[B13] Mason RJ Lee JM Curran JM Moss A Van Der Heide B Daniels PW Serological survey for *Ehrlichia canis* in urban dogs from the major population centres of northern Australia Aust Vet J 2001 79 8 559 562 10.1111/j.1751-0813.2001.tb10749.x 11599818

[B14] Parola P Roux V Camicas JL Baradji I Brouqui P Raoult D Detection of *Ehrlichiae* in African ticks by polymerase chain reaction Trans R Soc Trop Med Hyg 2000 94 6 707 708 10.1016/S0035-9203(00)90243-8 11198664

[B15] Baneth G Kenny MJ Tasker S Anug Y Shkap V Levy A Shaw SE Infection with a proposed new subspecies of *Babesia canis, Babesia canis* subsp. *presentii,* in domestic cats J Clin Microbiol 2004 42 1) 99 105 14715738 10.1128/JCM.42.1.99-105.2004 PMC321699

[B16] Tasker S Peters IR Mumford AD Day MJ Gruffydd-Jones TJ Day S Pretorius A-M Birtles RJ Helps CR Neimark H Investigation of human haemotropic *Mycoplasma* infections using a novel generic haemoplasma qPCR assay on blood samples and blood smears J Med Microbiol 2010 59 11 1285 1292 10.1099/jmm.0.021691-0 20651038 PMC3090618

[B17] Criado-Fornelio A Martinez-Marcos A Buling-Sarana A Barba-Carretero JC Presence of *Mycoplasma haemofelis, Mycoplasma haemominutum* and piroplasmids in cats from southern Europe: a molecular study Vet Microbiol 2003 93 4 307 317 10.1016/S0378-1135(03)00044-0 12713893

[B18] Altschul SF Gish W Miller W Myers EW Lipman DJ Basic local alignment search tool J Mol Biol 1990 215 3 403 410 2231712 10.1016/S0022-2836(05)80360-2

[B19] Saitou N Nei M The neighbor-joining method: a new method for reconstructing phylogenetic trees Mol Biol Evol 1987 4 4 406 425 3447015 10.1093/oxfordjournals.molbev.a040454

[B20] Kimura M A simple method for estimating evolutionary rates of base substitutions through comparative studies of nucleotide sequences J Mol Evol 1980 16 2 111 120 10.1007/BF01731581 7463489

