# Supplementary material for: Haemoparasites of free-roaming dogs associated with several remote Aboriginal communities in Australia
Source: BMC Vet Res. 2012 May 14;8:55. doi: 10.1186/1746-6148-8-55 (PMC3489665; doi:10.1186/1746-6148-8-55)
Supplement: Additional file 1 — Table S1. For individual dogs: population data (Aboriginal community; age; sex); Babesia vogeli (Bv) conventional PCR (cPCR) results [positive or not-detected (n/d)], Anaplasma platys (Ap), ‘Candidatus Mycoplasma haematoparvum’ (CMhp) and Mycoplasma haemocanis (Mhc) quantitative PCR (qPCR) results [threshold cycle for positive samples or not-detected (n/d)]; Dirofilaria immitis (Di), Borrelia burgdorferi (Bb), Ehrlichia canis (Ec), and A. platys (Ap) serological results. [file 1746-6148-8-55-S1.doc]

**Supplementary Table 1**: For individual dogs: population data (Aboriginal community; age; sex); *Babesia vogeli* (Bv) conventional PCR (cPCR) results [positive or not-detected (n/d)], *Anaplasma platys* (Ap), *‘Candidatus* Mycoplasma haematoparvum’ (CMhp) and *Mycoplasma haemocanis* (Mhc) quantitative PCR (qPCR) results [threshold cycle for positive samples or not-detected (n/d)]; *Dirofilaria immitis* (Di), *Borrelia burgdorferi* (Bb), *Ehrlichia canis* (Ec), and *A. platys* (Ap) serological results.

|  | **Population** | | | **PCR** | | | | **Serology** | | | |
| --- | --- | --- | --- | --- | --- | --- | --- | --- | --- | --- | --- |
| **ID** | **Community** | **Age** | **Sex** | **Bv cPCR** | **Ap qPCR** | **CMhp qPCR** | **Mhc qPCR** | **Di** | **Bb** | **Ec** | **Ap** |
| 1 | Ti Tree (Nturiya) | adult | male | n/d | n/d | 35.2 | 24.7 | n/d | n/d | n/d | n/d |
| 2 | Ti Tree (Nturiya) | adult | male | n/d | 40.6 | 28.6 | n/d | n/d | n/d | n/d | n/d |
| 3 | Ti Tree (Nturiya) | adult | female | n/d | 38.4 | n/d | n/d | positive | n/d | n/d | positive |
| 4 | Ti Tree (Nturiya) | adult | female | n/d | 34.9 | n/d | n/d | n/d | n/d | n/d | n/d |
| 5 | Ti Tree (Nturiya) | adult | male | n/d | n/d | n/d | 22.3 | n/d | n/d | n/d | n/d |
| 6 | Ti Tree (Nturiya) | juvenile | female | positive | 35.0 | n/d | n/d | n/d | n/d | n/d | n/d |
| 7^†^ | Ti Tree (Nturiya) | juvenile | male | n/d | n/d | n/d | n/d | n/d | n/d | n/d | n/d |
| 8 | Ti Tree (Nturiya) | juvenile | male | n/d | n/d | n/d | 24.4 | n/d | n/d | n/d | n/d |
| 9 | Ti Tree (Nturiya) | adult | male | n/d | n/d | n/d | n/d | n/d | n/d | n/d | n/d |
| 10 | Ti Tree (Nturiya) | adult | male | n/d | n/d | 30.3 | 29.6 | positive | n/d | n/d | n/d |
| 11 | Ti Tree (Nturiya) | adult | male | n/d | n/d | n/d | 24.9 | n/d | n/d | n/d | n/d |
| 12 | Ti Tree (Pmara) | juvenile | female | n/d | n/d | n/d | n/d | n/d | n/d | n/d | n/d |
| 13 | Ti Tree (Pmara) | adult | female | n/d | n/d | 31.3 | 38.1 | n/d | n/d | n/d | n/d |
| 14 | Ti Tree (Pmara) | adult | male | n/d | n/d | 29.5 | 28.9 | n/d | n/d | n/d | n/d |
| 15 | Ti Tree (Pmara) | adult | male | n/d | n/d | 31.3 | 27.9 | n/d | n/d | n/d | positive |
| 16 | Ti Tree (Pmara) | adult | female | n/d | 35.1 | n/d | 37.2 | n/d | n/d | n/d | positive |
| 17 | Ti Tree (Pmara) | adult | female | n/d | 41.3 | n/d | 33.0 | n/d | n/d | n/d | positive |
| 18 | Ti Tree (Pmara) | adult | male | positive | n/d | n/d | 23.2 | n/d | n/d | n/d | positive |
| 19 | Ti Tree (Pmara) | juvenile | male | n/d | n/d | n/d | 24.5 | n/d | n/d | n/d | n/d |
| 20 | Ti Tree (Pmara) | adult | female | n/d | n/d | n/d | 27.7 | n/d | n/d | n/d | n/d |
| 21 | Ti Tree (Pmara) | adult | female | positive | n/d | n/d | n/d | n/d | n/d | n/d | n/d |
| 22 | Ti Tree (Pmara) | puppy | male | positive | 40.1 | 33.0 | 38.2 | n/d | n/d | positive | n/a |
| 23 | Tiwi | adult | female | positive | 35.2 | n/d | 28.4 | n/d | n/d | n/d | n/d |
| 24 | Tiwi | adult | female | positive | 39.4 | n/d | n/d | n/d | n/d | n/d | n/d |
| 25 | Tiwi | juvenile | male | positive | 27.3 | n/d | n/d | n/d | n/d | n/d | positive |
| 26 | Goodooga | adult | female | positive | n/d | n/d | n/d | n/d | n/d | n/d | n/d |
| 27 | Goodooga | adult | female | n/d | n/d | n/d | n/d | n/d | n/d | n/d | n/d |
| 28 | Goodooga | puppy | male | positive | 24.5 | n/d | 29.3 | n/d | n/d | n/d | n/d |
| 29 | Goodooga | adult | male | positive | 29.4 | 27.2 | n/d | n/d | n/d | n/d | n/d |
| 30 | Goodooga | adult | female | n/d | n/d | n/d | n/d | n/d | n/d | n/d | n/d |
| 31 | Bidyadanga | adult | male | positive | 38.9 | n/d | n/d | n/d | n/d | n/d | positive |
| 32 | Bidyadanga | adult | male | n/d | n/d | n/d | 26.0 | n/d | n/d | n/d | positive |
| 33^†^ | Bidyadanga | puppy | male | positive | 33.4 | n/d | n/d | n/d | n/d | n/d | n/d |
| 34 | Bidyadanga | juvenile | female | positive | 32.6 | n/d | n/d | n/d | n/d | n/d | n/d |
| 35 | Bidyadanga | puppy | female | positive | n/d | n/d | n/d | n/d | n/d | n/d | positive |
| 36 | Bidyadanga | adult | female | n/d | 31.7 | n/d | n/d | n/d | n/d | n/d | n/d |
| 37 | Bidyadanga | puppy | male | positive | 30.8 | n/d | n/d | n/d | n/d | n/d | n/d |
| 38 | Bidyadanga | puppy | female | positive | 24.4 | n/d | n/d | n/d | n/d | n/d | n/d |
| 39 | Bidyadanga | puppy | female | positive | 27.7 | n/d | n/d | n/d | n/d | n/d | n/d |

^†^The novel haemoplasma was detected in these dogs
